# High levels of expression of the transcription factor Brachyury induce resistance of human carcinoma cells to immune-mediated attack

**DOI:** 10.1186/2051-1426-1-S1-P152

**Published:** 2013-11-07

**Authors:** Duane H Hamilton, Bruce Huang, Romaine I Fernando, Kwong-Yok Tsang, Claudia Palena

**Affiliations:** 1 Laboratory of Tumor Immunology and Biology, Center for Cancer Research, NCI, NIH, Bethesda, MD, USA

## 

The phenomenon of epithelial-mesenchymal transition (EMT) is a phenotypic switch that allows epithelial tumor cells to acquire features of mesenchymal cells, including the ability to migrate and invade and, therefore, to disseminate from the primary site. In addition to promoting tumor dissemination, various reports have demonstrated that acquisition of features of EMT associates with tumor resistance to the cytotoxic effects of chemotherapy, radiation, and some targeted therapies. It is not well understood, however, the contribution of EMT to the escape of tumors from host immune-surveillance and immune-mediated rejection. Our laboratory has characterized the T-box transcription factor Brachyury as a tumor-associated antigen and a regulator of EMT in human carcinomas. Currently, a Brachyury-based, recombinant yeast cancer vaccine is ongoing Phase I clinical testing for the treatment of patients with advanced carcinomas. In the present study, human epithelial carcinoma cell lines undergoing EMT via Brachyury over-expression and their epithelial tumor cell counterparts were comparatively utilized to evaluate whether EMT imparts resistance to immune-mediated attack. Our results demonstrate a bell-shaped relationship between the levels of Brachyury expression and the susceptibility of human carcinoma cell lines to lysis by immune cells. While tumor cells with low/intermediate levels of Brachyury expression are vulnerable to cell lysis, those with high levels of Brachyury expression are very resistant to lysis by immune cells. Our results demonstrate that a high level of Brachyury expression in human tumor cells reduces their susceptibility to antigen-specific CD8+ cytotoxic T cells and innate, natural killer (NK) and lymphokine-activated killer (LAK) cells. The mechanisms involved in this phenomenon are currently under investigation, and our results indicate that targeting the process of EMT may be a successful strategy not only to interfere with tumor dissemination but also to increase the effectiveness of immunotherapeutic interventions against metastatic tumors.

